# Network pharmacology analysis and experimental validation to explore the mechanism of Bushao Tiaozhi capsule (BSTZC) on hyperlipidemia

**DOI:** 10.1038/s41598-022-11139-2

**Published:** 2022-04-28

**Authors:** Guanlin Xiao, Zhihao Zeng, Jieyi Jiang, Aili Xu, Sumei Li, Yangxue Li, Zhao Chen, Weitao Chen, Jingnian Zhang, Xiaoli Bi

**Affiliations:** 1 Guangdong Province Engineering and Technology Research Institute of Traditional Chinese Medicine/Guangdong Provincial Key Laboratory of Research and Development in Traditional Chinese Medicine, Guangzhou, 510095 China; 2 School of the Fifth Clinical Medicine, Guangzhou University of Chinese Medicine, Guangzhou, 510405 China

**Keywords:** Computational biology and bioinformatics, Diseases, Medical research

## Abstract

Bushao Tiaozhi Capsule (BSTZC) is a novel drug in China that is used in clinical practice and has significant therapeutic effects on hyperlipidemia (HLP). In our previous study, BSTZC has a good regulatory effect on lipid metabolism of HLP rats. However, its bioactive compounds, potential targets, and underlying mechanism remain largely unclear. We extracted the active ingredients and targets in BSTZC from the Traditional Chinese Medicine Systems Pharmacology Database and Analysis Platform (TCMSP) and literature mining. Subsequently, core ingredients, potential targets, and signaling pathways were determined through bioinformatics analysis, including constructed Drug-Ingredient-Gene symbols-Disease (D-I-G-D), protein–protein interaction (PPI), the Gene Ontology (GO), and the Kyoto Encyclopedia of Genes and Genomes (KEGG). Finally, the reliability of the core targets was evaluated using in vivo studies. A total of 36 bioactive ingredients and 209 gene targets were identified in BSTZC. The network analysis revealed that quercetin, kaempferol, wogonin, isorhamnetin, baicalein and luteolin may be the core ingredients. The 26 core targets of BSTZC, including IL-6, TNF, VEGFA, and CASP3, were considered potential therapeutic targets. Furthermore, GO and KEGG analyses indicated that the treatment of HLP by BSTZC might be related to lipopolysaccharide, oxidative stress, inflammatory response and cell proliferation, differentiation and apoptosis. The pathway analysis showed enrichment for different pathways like MAPK signaling pathway, AGE-RAGE signaling pathway in diabetic, IL-17 signaling pathway and TNF signaling pathway. In this study, network pharmacology analysis, and experiment verification were combined, and revealed that BSTZC may regulate key inflammatory markers and apoptosis for ameliorating HLP.

## Introduction

Hyperlipidemia (HLP) is a major risk factor for different cardiovascular diseases (CVD_S_), type II diabetes mellitus, hypertension, and atherosclerosis^1,2^. The concentrations of lipids, such as triglycerides (TG), total cholesterol (TC) and low-density lipoprotein (LDL) increase, or the levels of high-density lipoprotein (HDL) decrease in the blood^3^.

Bushao Tiaozhi Capsule (BSTZC) is composed of Microctis Folium (BZY, *Microcos paniculata L.*), Paeoniae Radix Rubra (CS, *Paeonia lactiflora Pall.*), Curcumae Rhizoma (EZ, *Curcuma phaeocaulis Val.*), and Andrographis Herba (CXL, *Andrographis paniculata* (*Burm.f*) *Nees*). Our previous clinical studies have shown that BSTZC was effective and relatively safe in the treatment of HLP, with no obvious adverse events. Compared with the rat models with HLP of group, BSTZC significantly reduced the serum TC, TG, LDL-C and ApoB levels, improved the HDL-C and ApoA1 levels and ApoA1/ApoB ratio, reduced the hepatic TC and TG levels and promoted hepatic LCAT and LXR-α gene expression (all *P* < 0.05). BSTZC has a good regulatory effect on lipid metabolism of HLP rats^4^. Moreover, the chemical profile of BSTZC has been fully investigated using a UPLC–TOF–MS/MS method, a total of 53 chemical constituents were identified by literature comparison, and high mass spectrometry data analysis. The chemical constituent cluster was composed of 21 flavonoids, 10 phenolics, 5 monoterpene glycosides, 7 diterpene lactones and 10 sesquiterpenes, the identified chemical components mostly cover the main constituents of each medicinal material in the formula^5^. BSTZC has a significant effect on HLP, similar to other TCM formulae, BSTZC involves multiple components, targets, and pathways treatment of HLP. However, its core active ingredients and their potential mechanism of action have not been fully elucidated.

Network pharmacology is the basis of constructing multilayer networks of disease-phenotype-gene-drug, revealing the scientific basis and therapeutic mechanism of TCM formulae^6–8^. With the popularization of bioinformatics and pharmacology, network pharmacology has been applied to drug design including the construction of disease networks, drug-target networks and drug-disease networks. This method can clearly observe the interaction between drugs and diseases, which is consistent with the TCM theory that emphasizes the synergistic effect of Chinese medicine^9^.

**Figure 1:**
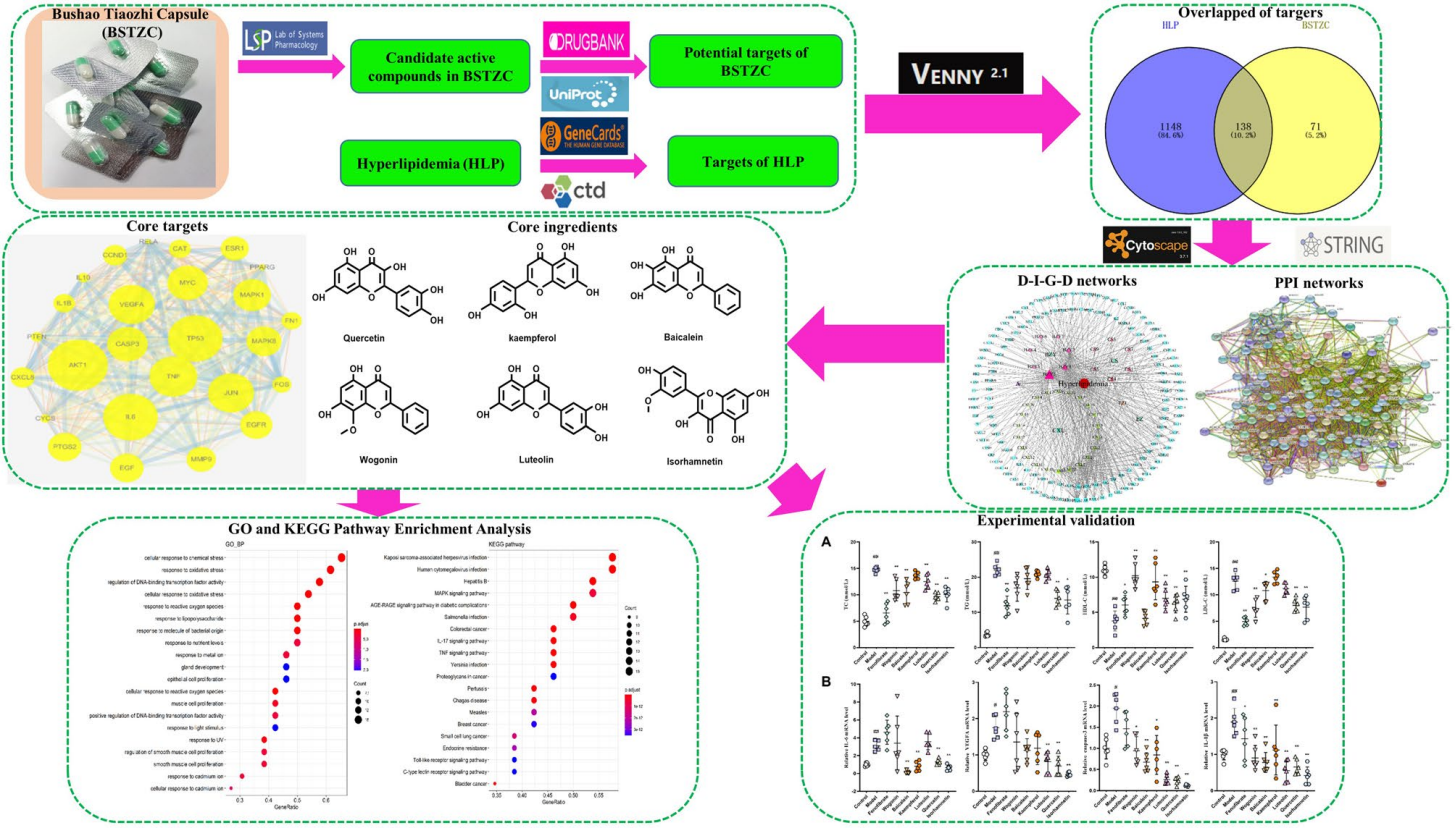
Workflow diagram of the network pharmacology-based analysis of BSTZC in the treatment of HLP.

Based on a network pharmacology and vivo experiment studies, this study aims to explore the potential mechanisms and pathways of the core active ingredients of BSTZC in the treatment of HLP. The workflow is shown in Fig. 1, our findings will provide a theoretical basis for the clinical application of BSTZC.

## Materials and methods

### Drugs and reagents

Triton WR-1339 was purchased from Sigma-Aldrich (Lot#MKCC6730; Shanghai, China). Fenofibrate was purchased from Abbott Laboratories Limited (Lot#27232). Quercetin (pure ≥ 97%, CAS No.: 117-39-5), kaempferol (pure ≥ 98%, CAS No.: 520-18-3), baicalein (pure ≥ 98%, CAS No.: 27462-75-5), isorhamnetin (pure ≥ 98%, CAS No.: 480-19-3), luteolin (pure ≥ 98%, CAS No.: 491-70-3), and wogonin (pure ≥ 98%, CAS No.: 632-85-9) were purchased from Ruifensi. Ltd.Chengdu, China.

### Animal models and ethics statements

All experiment procedures were compiled with the NIH recommendations for the use and care of animals. The animal experimental protocols were reviewed and approved by the animal ethics committee of Guangdong Provincial Engineering Technology Institute of Traditional Chinese Medicine (Guangzhou, China), and all animal experiments were performed in accordance with relevant ARRIVE guidelines^10^. The 54 male C57BL/6 mice weighing between 18 and 22 g were obtained from the Guangdong Medical Laboratory Animal Center (Guangzhou, China). All the mice were fed normal diet and housed in barrier system at standard room temperature and a 12 h light/dark cycle conditions. The experiment mice were divided into the following nine groups: control group, model group, fenofibrate group (26 mg/kg), wogonin group (25 mg/kg), baicalein group (25 mg/kg), kaempferol group (25 mg/kg), luteolin group (25 mg/kg), quercetin group (25 mg/kg) and isorhamnetin group (25 mg/kg). The administration groups were given corresponding drugs by gavage, once a day, for 5 days. On the third day of administration, except for the normal control group, all the mice in the other groups were intramuscularly injected with triton-1339 (480 mg/kg, i.m.) to induce acute hyperlipidemia model. At the end of the experimental period, all mice were anesthetized using isoflurane and sacrificed by inner canthus artery exsanguination, then the organs were reserved for analysis.

### Collection and screening of candidate active compounds in BSTZC

TCMSP (https://tcmspw.com/tcmsp.php) and literature mining were searched to collect the related active compounds of BSTZC. Oral bioavailability (OB) is one of the most important pharmacokinetic parameters, it represents the ability of a drug to enter the circulation. Drug-likeness (DL) indicates the similarity between a molecule and known drugs. OB and DL were used as the main parameters to screen the active ingredients, according to absorption, distribution, metabolism, excretion (ADME) criteria OB ≥ 30% and DL ≥ 0.18 were screened for eligible ingredients^11–13^.

### Prediction of potential targets of BSTZC

TCMSP database was applied in this work. Related targets (DrugBank database, https://go.drugbank.com/) of active components of BSTZC were predicted on TCMSP platform and transformed the target name to standard gene name on Uniport (https://www.uniprot.org/) database, and then removed the duplications.

### Identification of associated targets of HLP

HLP related genes were collected from CTD (http://ctdbase.org/) and GeneCards (https://www.genecards.org/) database, with “hyperlipidemias” and “hyperlipidemia” as keyword. The genes from the above databases merged and removed the duplications.

### D-I-G-D network construction and analysis

Venny 2.1.0 (https://bioinfogp.cnb.csic.es/tools/venny/) was used to find out the overlapped targets between compound targets and disease targets. To explore the relationship between D-I-G-D more reasonably, Cytoscape 3.7.1 (https://cytoscape.org/) with visualized tool and all node degrees of networks were calculated. The color and node size scale were used to explain the whole network based on the number of edges. The node with the maximum number of edge count was indicated with large node.

### Protein–protein interaction (PPI) network construction and analysis

Overlapping targets of compound -disease were added into STRING (https://string-db.org/), the screening condition used was “*Homo sapiens*”, and the results were saved. The resulting file was imported into Cytoscape v3.7.1 software, and BisoGenet and CytoNCA plug-in in Cytoscape were used to calculate the degree centrality (DC), betweenness centrality (BC), and closeness centrality (CC). The core target of the protein–protein interaction (PPI) network was filtered^14^.

### Gene ontology (GO) enrichment and Kyto Encyclopedia of Genes and Genomes (KEGG) pathway analysis

We imported the core targets on the Bioconductor ClusterProfiler, org.Hs.eg.db and DOSE packages of R 4.0.2 (https://cran.r-project.org/src/base/R-4/) software to conduct conducted the Gene Ontology (GO) biological process enrichment analysis and Kyoto Encyclopedia of Genes and Genomes data obtained (KEGG) pathway enrichment with *p* < 0.05 and q < 0.05 as the thresholds^14–17^.

### Lipid analysis

Plasma samples of animal experiments were obtained by centrifuge at 3000 rpm at 4 °C for 10 min and preserved at −80 °C before analysis. TC, TG, HDL-C and LDL-C levels of serum were measured by Microplate Reader (Varioskan Flash, Thermo, USA) with commercial kits from Nanjing Jiancheng (Jiangsu, China).

### The quantitative real-time PCR (RT-qPCR)

Liver samples of animal experiments were obtained by mechanically homogenized in ice water bath and centrifuged for 10 min at 2500 rpm, then the supernatant was collected. Total RNA from liver tissues was extracted using Trizol reagent (Dingguo Changsheng, Beijing, China) and reverse transcribed. RT-qPCR reactions was performed on iQ5 Multicolor Real-Time PCR detection system (BIO-RAD, Hercules, California, USA) with SYBR Green Dye detection (TaKaRa Bio, Kusatsu, Japan). The data were analyzed using the 2^−ΔΔCt^ method, with 18 s as a reference in the mRNA analysis. The primers were shown in Table 1.

**Table 1:** The primer sequences for mRNA in RT-qPCR.

Primer	Sequences
*mIl-1β*	Sense: GAAATGCCACCTTTTGACAGTG Anti-sense:* CTGGATGCTCTCATCAGGACA*
*mCaspase3*	Sense: CTGACTGGAAAGCCGAAA Anti-sense: AAAGGGACTGGATGAACC
*mIl-6*	Sense: ATCTCACCAATGACCGCTAT Anti-sense: CTGCTGCCAGTCTTCAACAC
*mVegfa*	Sense: *GCACATAGGAGAGATGAGCTTCC* Anti-sense: *CTCCGCTCTGAACAAGGCT*
*m18s*	Sense: *ACGGCTACCACATCC* Anti-sense: *CAGACTTGCCCTCCA*

### Statistical analysis

All the grouped data were statistically evaluated with the SPSS 22.0 software. Kruskal–Wallis test and one-way ANOVA followed by LSD post hoc test were used to determine the significance of the differences between the groups. The value of *p* < 0.05 was considered statistically significant. All the results were expressed as mean ± SEM.

## Results

### Active ingredients and putative targets of BSTZC

On the basis of TCMSP and literature mining^18–20^, the molecular structure of each active compound was confirmed by TCMSP database. According to the screening threshold (OB ≥ 30%, DL ≥ 0.18), BSTZC was found to be comprised of 43 compounds (Supplementary Table S1). These active compounds were found in BZY (8 compounds), CS (13 compounds), EZ (1 compounds), CXL (22 compounds). The component targets of BSTZC predicted by TCMSP database were obtained through gene annotation in Uniprot database. Moreover, 209 predicted targets of BSTZC were found based on the obtained compounds.

### Targets of HLP

In the CTD database, we screened 785 targets among 24,808 genes related to HLP, and the screening threshold was “Inference score” ≥ 50.45. Meanwhile, 688 targets were obtained among from 1373 genes on GeneCards with “Relevance score” ≥ 1.57 as the screening threshold. Subsequently, the targets in the above databases were merged and removed the overlapped targets, a total of 1286 targets of HLP were finally obtained.

### D-I-G-D network

**Figure 2:**
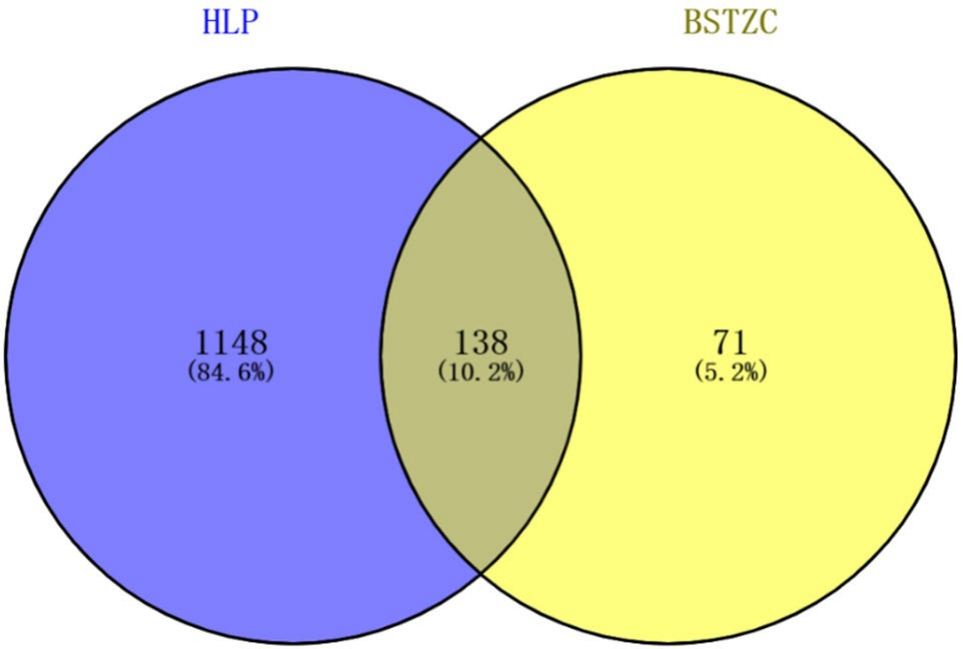
Venn diagram of related targets of BSTZC and HLP.

**Figure 3:**
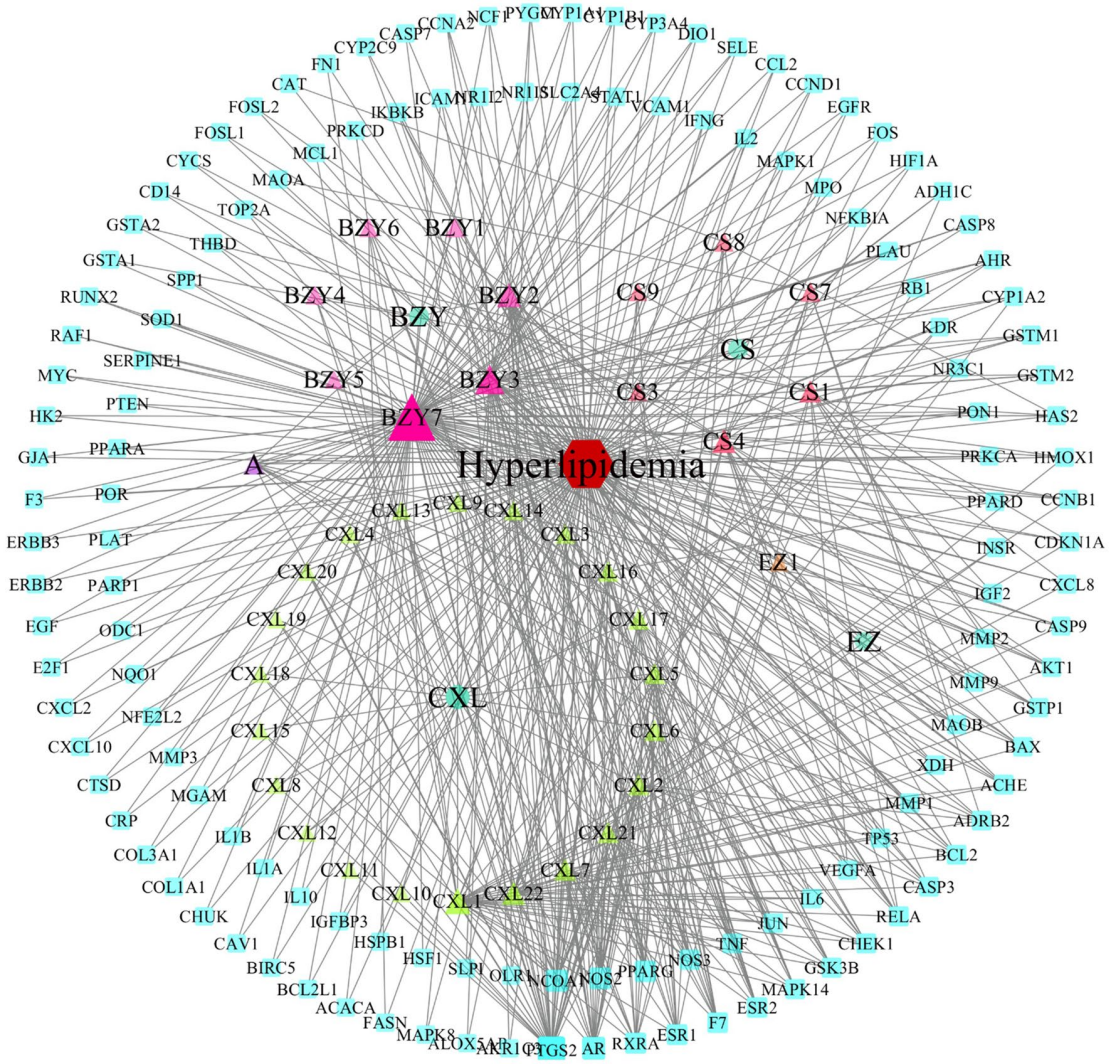
The Drug-Ingredients-Gene symbols-Disease (D-I-G-D) network of BSTZC.

**Figure 4:**
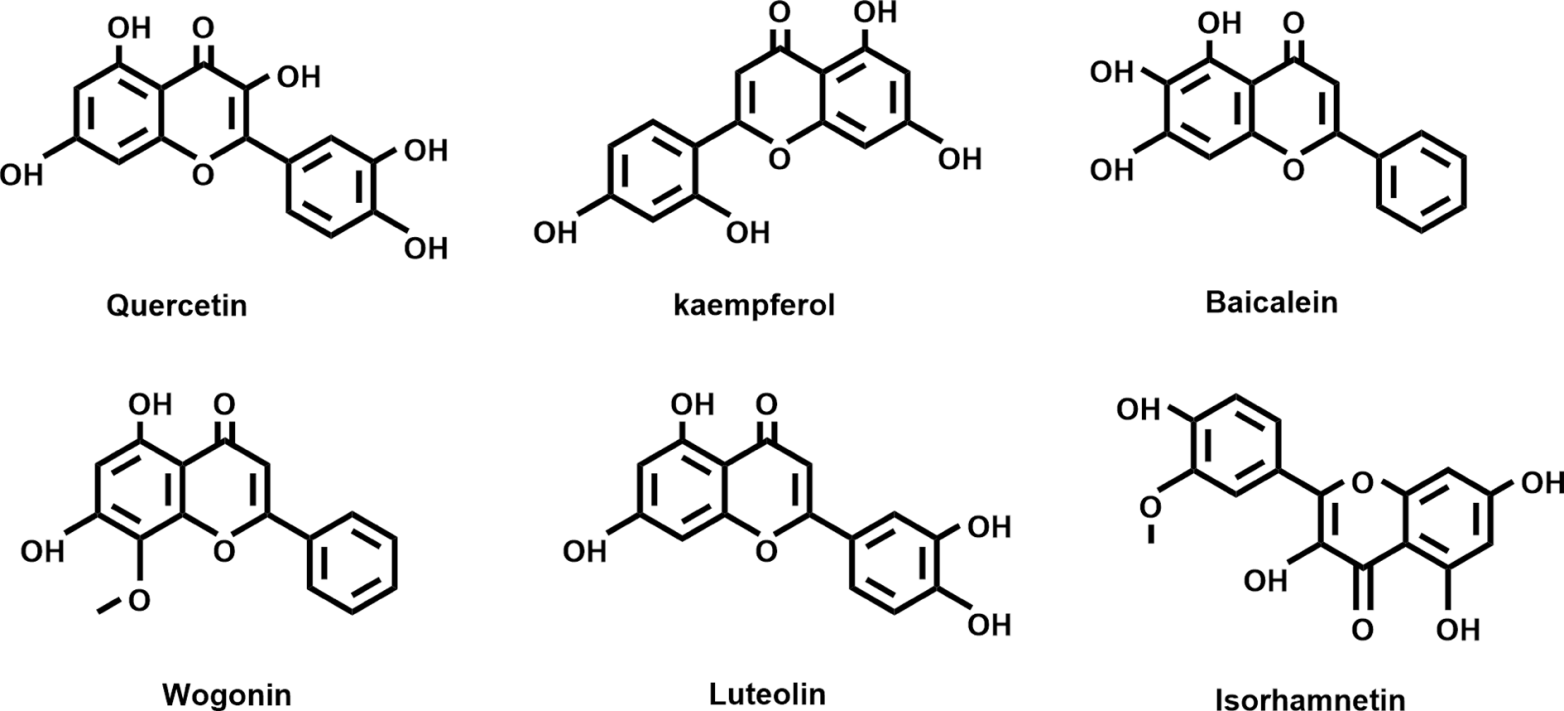
Chemical structures of the 6 core active ingredients.

After removing the duplicate targets, a total of 209 drug-targets and 1286 disease-targets were obtained. The VENNY 2.1 software was used to cross disease-related targets with drug-related targets, and to create the disease-drug overlapping targets Venny diagram (Fig. 2), 138 overlapping targets were obtained. The network diagram of D-I-G-D for the treatment of HLP with BSTZC (Cytoscape 3.7.1) was drawn (Fig. 3). The red hexagon represents the disease, the green round represents the four herbs of BSTZC, the triangle represents the compound, and the aqua rectangle represents the target. The larger the node, the darker the color, the greater the degree value in the network, indicating greater importance. The network map included a total of 180 nodes, 587 edges, including 138 target gene nodes and 37 active component nodes (8 BZY, 7 CS, 1 EZ, 22 CXL), among which beta-sitosterol was the common compound of BZY and CS. Then, we analyzed and reordered these compounds in descending order of degree, higher degrees indicated that the ingredients play more important anti-HLP roles. Six compounds were considered to have high connection with potential targets of HLP as follows: quercetin (degree = 109), kaempferol (degree = 42), wogonin (degree = 30), isorhamnetin (degree = 22), baicalein (degree = 21), luteolin (degree = 20), suggested these components may be the core active ingredients of BSTZC anti-HLP (Fig. 4).

### PPI network analysis

**Figure 5:**
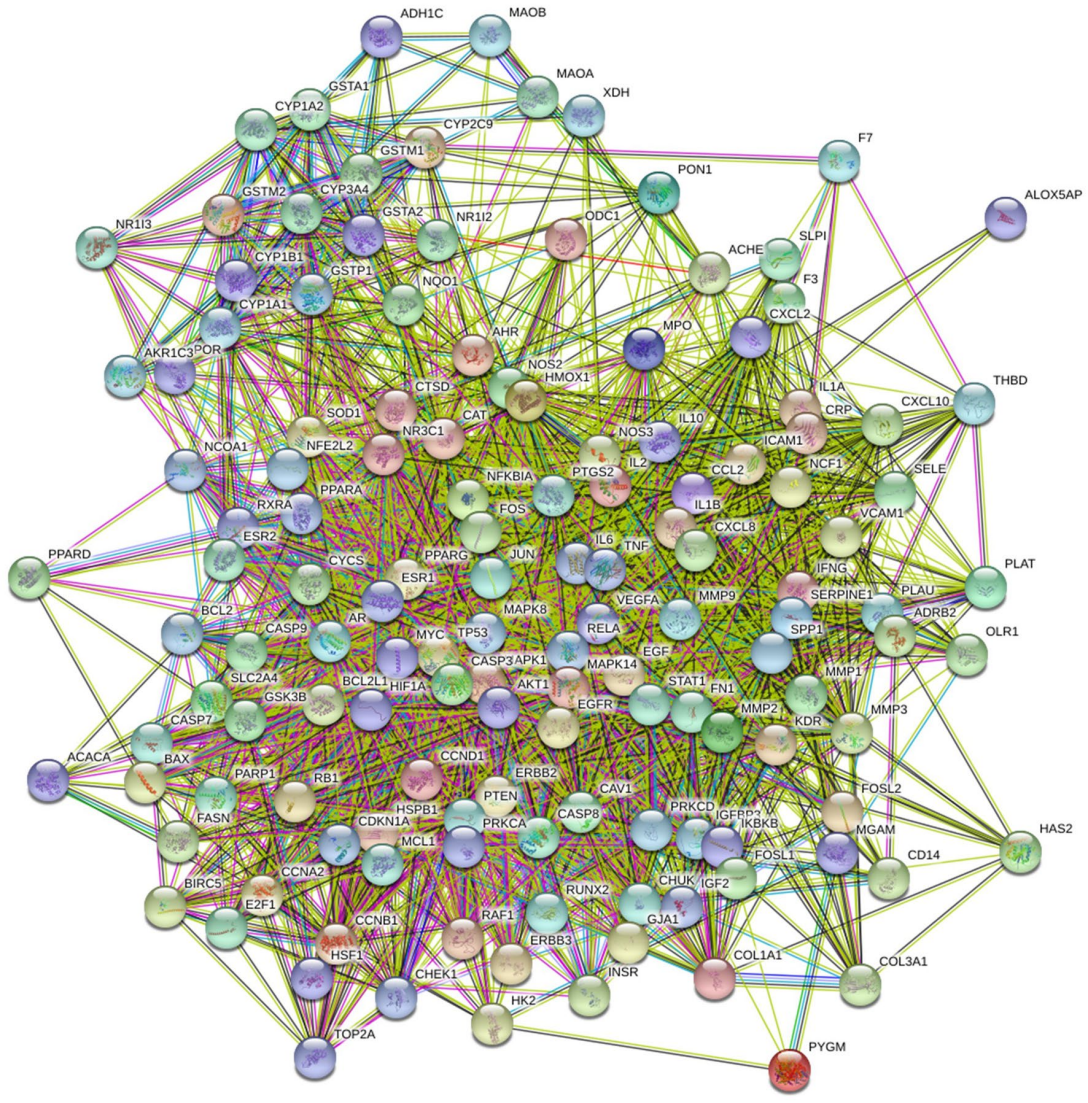
PPI network compound targets against HLP. The original PPI data generated from the STRING database showing the detailed interactions of the targets.

**Figure 6:**
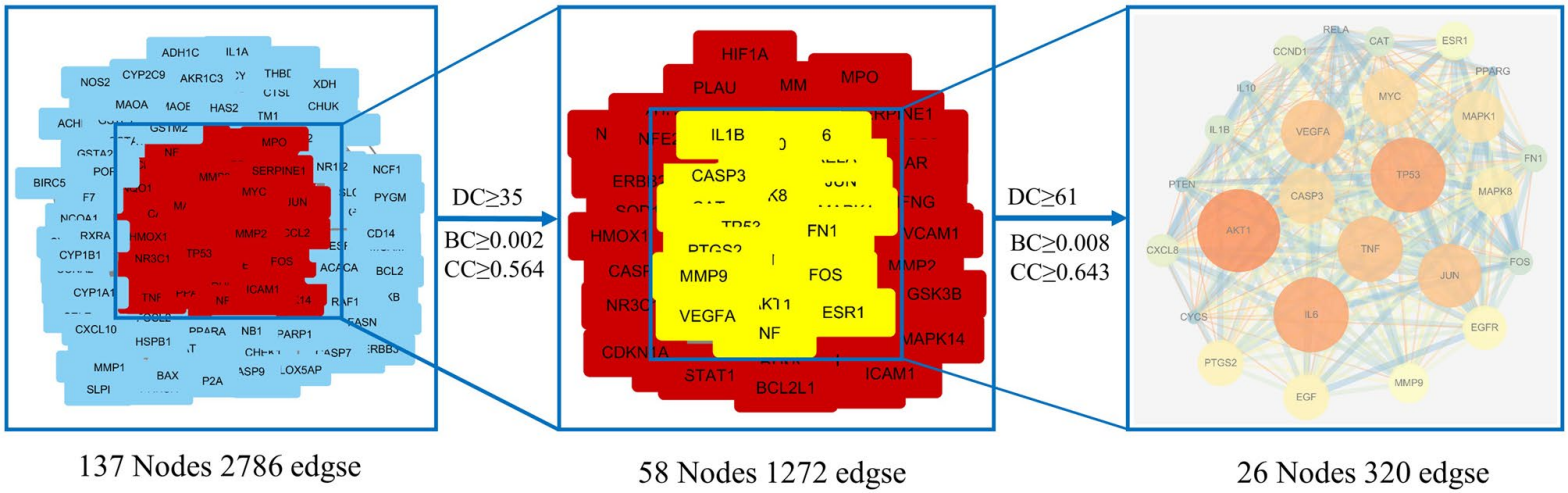
The whole screening process for the PPI network through a topological method. In the third image, the bigger size and brighter color represent higher DC value.

To construct a PPI network consisting of 137 nodes and 2786 edges, a total of 138 disease-drug overlapping targets were introduced into STRING. According to the results obtained from the STRING, there were 138 relevant target proteins (1 target proteins were removed because they did not interact with other proteins) (Fig. 5). PPI network diagrams were imported into Cytoscape software for visualization (Fig. 6). Then, three main parameters of Degree (DC), Betweenness Centrality (BC), and Closeness Centrality (CC) were used to select the key genes and construct the major hub nodes for the anti-HLP effect of BSTZC. The first screening threshold was DC ≥ 35, BC ≥ 0.002 and CC ≥ 0.564, which resulted in 58 nodes and 1272 edges. Subsequently, these 58 key nodes were further screened with the second threshold of DC ≥ 61, BC ≥ 0.008 and CC ≥ 0.643, and 26 nodes and 320 edges remained. After screening according to the three main parameters, the network indicated that these 26 genes play a key role in treatment of HLP, the node became larger and its color changed from yellow to red with the increased degree of the targets. The 26 targets were selected as the core targets as followed: AKT1, IL-6, TP53, TNF, VEGFA, JUN, MYC, CASP3, MAPK1, MAPK8, EGF, PTGS2, EGFR, MMP9, ESR1, CXCL8, CCND1, CAT, IL-1β, FN1, FOS, IL10, PTEN, CYCS, PPARG, and RELA (Table 2).

**Table 2:** The information of 26 core targets.

No	Description	Gene symbol	Degree	Uniprot ID
1	RAC-alpha serine/threonine-protein kinase	AKT1	102	207
2	Interleukin-6	IL6	98	3569
3	Cellular tumor antigen p53	TP53	98	7157
4	Tumor necrosis factor	TNF	93	7124
5	Vascular endothelial growth factor A	VEGFA	92	7422
6	Transcription factor AP-1	JUN	92	3725
7	Myc proto-oncogene protein	MYC	88	4609
8	Caspase-3	CASP3	88	836
9	Mitogen-activated protein kinase 1	MAPK1	86	5594
10	Mitogen-activated protein kinase 8	MAPK8	84	5599
11	Pro-epidermal growth factor	EGF	83	1950
12	Prostaglandin G/H synthase 2	PTGS2	83	5743
13	Epidermal growth factor receptor	EGFR	82	1956
14	Matrix metalloproteinase-9	MMP9	80	4318
15	Estrogen receptor	ESR1	79	2099
16	Interleukin-8	CXCL8	76	3576
17	G1/S-specific cyclin-D1	CCND1	76	595
18	Catalase	CAT	74	847
19	Interleukin-1 beta	IL-1β	74	3553
20	Fibronectin	FN1	73	2335
21	Proto-oncogene c-Fos	FOS	72	2353
22	Interleukin-10	IL10	66	3586
23	Phosphatidylinositol 3,4,5-trisphosphate 3-phosphatase and dual-specificity protein phosphatase PTEN	PTEN	66	5728
24	Cytochrome c	CYCS	65	54205
25	Peroxisome proliferator-activated receptor gamma	PPARG	63	5468
26	Transcription factor p65	RELA	62	5970

### GO and KEGG pathway enrichment analysis

**Figure 7:**
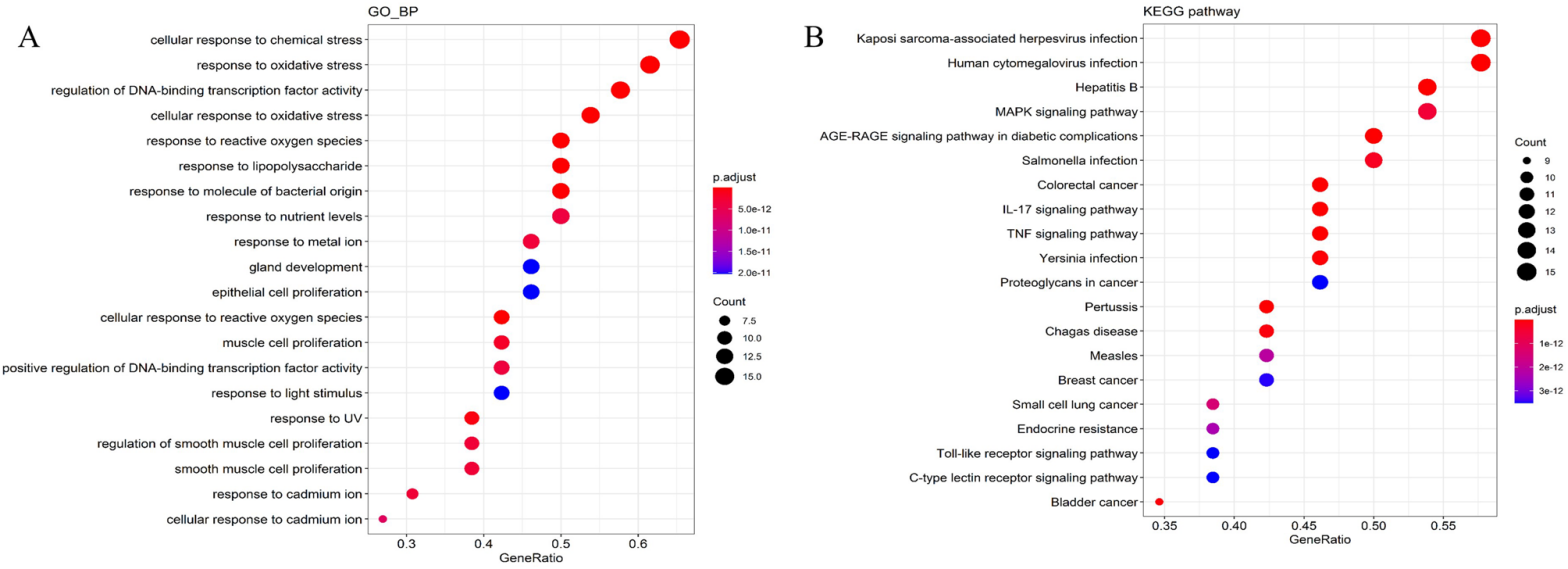
**(A)** Bubble chart of gene ontology (GO) function enrichment of core targets. **(B)** Bubble chart of Kyoto Encyclopedia of Genes and Genomes (KEGG) enrichment of core targets.

**Figure 8:**
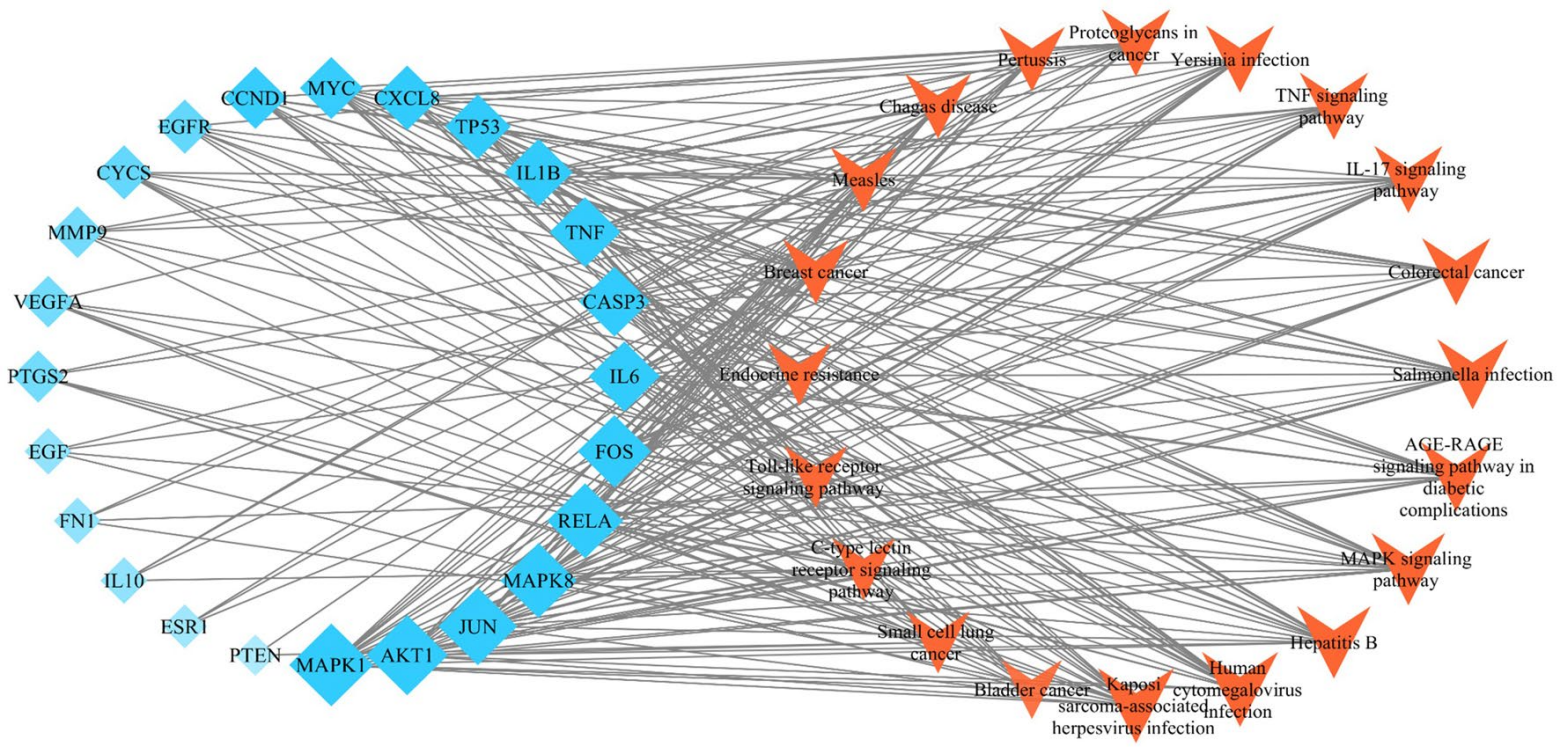
The targets-pathway network of BSTZC for treating HLP.

To further investigate the effector mechanism of BSTZC in the treatment of HLP, the 26 core target genes screened in PPI network were analyzed by GO biological process enrichment and KEGG pathway^21^. In total, there were 1715 GO biological process and 145 KEGG pathway enrichment results. The results showed that GO biological processes were related to the treatment of HLP, and included response to: cellular response to chemical stress(GO:0062197), response to oxidative stress (GO:0006979), regulation of DNA-binding transcription factor activity (GO:0051090), cellular response to oxidative stress(GO:0034599), response to reactive oxygen species(GO:0000302), and response to lipopolysaccharide (LPS) (GO:0032496). The bubble chart of the top 20 significant enrichment results in the GO analysis is shown in Fig. 7A (Supplementary Table S2). The 26 core targets were closely related to signaling pathways, such as MAPK signaling pathway (hsa04010), AGE-RAGE signaling pathway in diabetic complications (hsa04933), IL-17 signaling pathway (hsa04657), and TNF signaling pathway (hsa04668). The first 20 representative signaling pathways are shown in Table 3, KEGG bubble chart is shown in Fig. 7B, these pathways may be key pathways for treating HLP. In order to further screen the core targets enriched in significant pathways, Cytoscape software was used to construct the target-pathway networks, as shown in Fig. 8. The orange V-shapes represents the signaling pathway, and the light blue diamond represents the target, the node became larger and its color changed from light to dark with the increased degree of the targets and signaling pathways. The networks revealed that BSTZC was associated with the treatment of HLP through multi-targets and multi-pathways, we speculated that the underlying mechanism of BSTZC was probably related to its regulation of several biological.

**Table 3:** Top 20 clusters with their representative enriched terms.

Catagory	Description	LogP	Count	Gene
hsa05167	Kaposi sarcoma-associated herpesvirus infection	−17.90	15	AKT1, IL6, TP53, VEGFA, JUN, MYC, CASP3, MAPK1, MAPK8, PTGS2, CXCL8, CCND1, FOS, CYCS, RELA
hsa05163	Human cytomegalovirus infection	−16.74	15	AKT1, IL6, TP53, TNF, VEGFA, MYC, CASP3, MAPK1, PTGS2, EGFR, CXCL8, CCND1, IL-1β, CYCS, RELA
hsa05161	Hepatitis B	−17.11	14	AKT1, IL6, TP53, TNF, JUN, MYC, CASP3, MAPK1, MAPK8, MMP9, CXCL8, FOS, CYCS, RELA
hsa04010	MAPK signaling pathway	−13.45	14	AKT1, TP53, TNF, VEGFA, JUN, MYC, CASP3, MAPK1, MAPK8, EGF, EGFR, IL-1β, FOS, RELA
hsa04933	AGE-RAGE signaling pathway in diabetic complications	−18.17	13	AKT1, IL6, TNF, VEGFA, JUN, CASP3, MAPK1, MAPK8, CXCL8, CCND1, IL-1β, FN1, RELA
hsa05132	Salmonella infection	−13.78	13	AKT1, IL6, TNF, JUN, MYC, CASP3, MAPK1, MAPK8, CXCL8, IL-1β, FOS, CYCS, RELA
hsa05210	Colorectal cancer	−17.07	12	AKT1, TP53, JUN, MYC, CASP3, MAPK1, MAPK8, EGF, EGFR, CCND1, FOS, CYCS
hsa04657	IL-17 signaling pathway	−16.58	12	IL6, TNF, JUN, CASP3, MAPK1, MAPK8, PTGS2, MMP9, CXCL8, IL-1β, FOS, RELA
hsa04668	TNF signaling pathway	−15.63	12	AKT1, IL6, TNF, JUN, CASP3, MAPK1, MAPK8, PTGS2, MMP9, IL1B, FOS, RELA
hsa05135	Yersinia infection	−14.83	12	AKT1, IL6, TNF, JUN, MAPK1, MAPK8, CXCL8, IL-1β, FN1, FOS, IL10, RELA
hsa05205	Proteoglycans in cancer	−12.42	12	AKT1, TP53, TNF, VEGFA, MYC, CASP3, MAPK1, EGFR, MMP9, ESR1, CCND1, FN1
hsa05133	Pertussis	−15.77	11	IL6, TNF, JUN, CASP3, MAPK1, MAPK8, CXCL8, IL-1β, FOS, IL10, RELA
hsa05142	Chagas disease	−14.29	11	AKT1, IL6, TNF, JUN, MAPK1, MAPK8, CXCL8, IL-1β, FOS, IL10, RELA
hsa05162	Measles	−12.78	11	AKT1, IL6, TP53, JUN, CASP3, MAPK8, CCND1, IL-1β, FOS, CYCS, RELA
hsa05224	Breast cancer	−12.51	11	AKT1, TP53, JUN, MYC, MAPK1, EGF, EGFR, ESR1, CCND1, FOS, PTEN
hsa05222	Small cell lung cancer	−12.98	10	AKT1, TP53, MYC, CASP3, PTGS2, CCND1, FN1, PTEN, CYCS, RELA
hsa01522	Endocrine resistance	−12.70	10	AKT1, TP53, JUN, MAPK1, MAPK8, EGFR, MMP9, ESR1, CCND1, FOS
hsa04620	Toll-like receptor signaling pathway	−12.43	10	AKT1, IL6, TNF, JUN, MAPK1, MAPK8, CXCL8, IL-1β, FOS, RELA
hsa04625	C-type lectin receptor signaling pathway	−12.43	10	AKT1, IL6, TNF, JUN, MAPK1, MAPK8, PTGS2, IL-1β, IL10, RELA
hsa05219	Bladder cancer	−14.58	9	TP53, VEGFA, MYC, MAPK1, EGF, EGFR, MMP9, CXCL8, CCND1

### Active ingredients of BSTZC ameliorated acute hyperlipidemia in triton-1339W-induced mice

**Figure 9:**
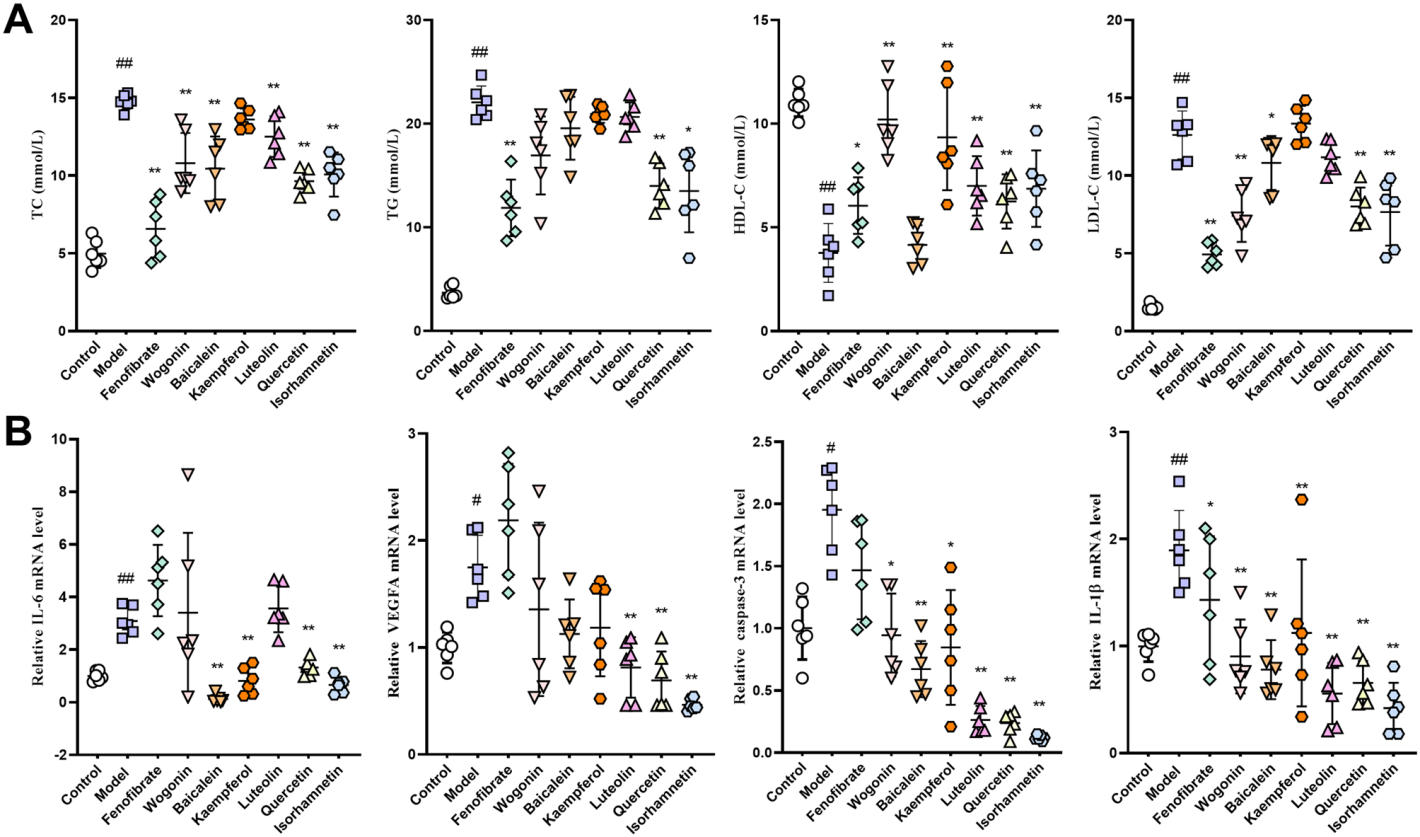
Effect of BSTZC on serum and liver in triton-1339W-induced HLP mice. **(A)** Biochemical analyses of serum TC, TG, HDL-C and LDL-C. **(B)** The expression of IL-6, VEGFA, caspase-3 and IL-1β mRNA level. The results are expressed as mean ± standard deviation. (n = 6). ^#^*P* < 0.05, ^##^*P* < 0.01 vs control group; *P < 0.05, **P < 0.01 vs model group.

To investigate the effect of core active ingredients on lipid metabolism and hepatoprotective, we analyzed the biochemical profiles of serum. The result was shown in Fig. 9A. Compared with the control group, serum TC, TG and LDL-C levels were obviously increased in the model groups. In contrast, serum HDL-C level was remarkably decreased in the model groups. Moreover, active ingredients of BSTZC (especially quercetin and isorhamnetin) remarkably decreased the level of serum TC, TG and LDL-C, which indicated that BSTZC was effective in ameliorating lipid metabolism disorder. Noticeably, compared with the model group, 5 active ingredients of BSTZC significantly increased serum HDL-C level which was the key lipoprotein for metabolism in RCT (reversal cholesterol transportation). It suggested that RTC was involved in the regulative mechanism of BSTZC.

### Active ingredients of BSTZC inhibited the gene expression of acute inflammation in triton-1339W-induced mice

Excessive lipid accumulation in liver was an important factor inducing the vascular inflammation. To further elucidate the mechanism of BSTZC on hyperlipidemia, the inflammation and apoptosis related genes were detected. Figure 9B was shown that relative to control groups, triton-1339W increased IL-6, VEGRA, IL-1β and caspase-3 mRNA expression. And compared with the model groups, IL-6, VEGRA, IL-1β and caspase-3 were decreased by core active ingredients. We thus infer that BSTZC may participate in the lipid metabolism progression through reduced inflammatory response and apoptosis.

## Discussion

In previous studies, it has been shown that BSTZC possessed beneficial effects in the treatment of HLP, however, due to multiingredient and multitarget characteristics of Chinese medicine, the specific underlying mechanism of the pharmacodynamic effects is still unclear. Therefore, a network pharmacology approach was used to investigate the underlying mechanism of action of BSTZC in the treatment of HLP. Based on the D-I-G-D network combined with literature studies, we found that quercetin, kaempferol, wogonin, isorhamnetin, baicalein, and luteolin played major roles in this network. Meanwhile, we found the six core active compounds were all flavonoids, which have been reported to have lipid-lowering, anti-inflammatory, and antioxidant effects^22–24^. We established triton-1339W-induced hyperlipidemia mice model, supplied with core active ingredients of BSTZC, in order to confirm its anti-HLP effect. The results showed that TC, TG, LDL-C were significantly reduced and HDL-C was significantly increased by the quercetin and isorhamnetin groups. The other four core active components also had a certain effect on lowering blood lipid level, which indicated that BSTZC can improve the lipid level of riton-1339W-induced hyperlipidemia mice. Quercetin has been studied for antioxidant, anti-inflammatory, lowering blood lipid, and anti-atherogenic properties effect, after quercetin treatment, the content of TC, TG, LDL and free fatty acid in HLP rats can be decreased^25,26^. Kaempferol is a flavonol that has been shown to regulate cellular lipid and glucose metabolism^27^. Isorhamnetin can protect vascular endothelial cells, inhibit adipose differentiation, inhibit vascular endothelial cell proliferation, anti-inflammatory and other effects. Moreover, it could significantly inhibited LPS-induced TNF-α, IL-1β and IL-6 secretion both in vitro and in vivo^28^. Baicalin had anti-inflammatory effects on chronic inflammation-related diseases such as CVDs and inflammatory bowel disease^29^. Luteolin can restore vascular endothelial NO availability in high fat diet mice, and prevent obesity-associated systemic metabolic alterations and vascular dysfunction through antioxidant and anti-inflammatory mechanisms^30^. Consequently, they may probably be core active compounds of BSTZC against HLP.

The PPI network showed that 26 core targets including IL-6, TNF, VEGFA, CASP3, and IL-1β were probably the most relevant targets for BSTZC in the treatment of HLP^31–33^. IL-6, VEGFA, TNF, and IL-1β are common proinflammatory factors. Overexpression of inflammatory mediators (such as cytokines and chemokines) leads to increased vascular permeability and a series of cascade reactions. Inflammatory responses can stimulate the oxidative stress response of adipocytes, affect their biological processes, such as proliferation and differentiation, and insulin sensitivity, and then lead to dyslipidemia and further accumulation of fat. IL-6, as a proinflammatory factor, affects the expression of other proinflammatory cytokines and chemokines in the early stage of inflammation, amplifying the inflammatory response^34,35^. IL-6 and IL-1β, as important mediators of inflammatory response, can aggravate dyslipidemia^36^. Vascular endothelial growth factor A (VEGFA) is an angiogenic factor in adipose tissue, and plays a key role in the regulation of angiogenesis in adipose tissue, when VEGFA is down-regulated expression, it inhibits adipose tissue angiogenesis, thereby reducing adipose tissue formation^37^. IL-6 could induce the up-regulation of VEGFA expression, accelerate the migration of vascular endothelial cells, increase vascular permeability, and induce angiogenesis^38^. TNF-α is an important regulator of inflammatory response and immune function^39^, which could induce the production of IL-1 and IL-6, and participate in systemic inflammatory response^40,41^. Furthermore, studies have shown that CASP3 plays a crucial role in the execution of cell apoptosis^42^. When HLP occurs, pancreatic cell damage activates CASP3 and leads to pancreatic cell apoptosis. At the same time, considering that HLP is often accompanied by fatty liver, oxidative stress increases the production of reactive oxygen species (ROS), leading to lipid peroxidation, and then leads to the production of TNF-α, IL-8 and other factors, which destroys the normal function of cells and leads to inflammation and apoptosis^43,44^. Enrichment analysis of GO and KEGG pathways on 26 core targets was performed, and we obtained 1715 GO biological processes and 145 KEGG pathways. Among them, GO function enrichment results were mostly related to oxidative stress response, ROS response, and lipopolysaccharide response. We speculated that the response to lipopolysaccharide and oxidative stress may be the most important biological process of BSTZC in the treatment of HLP. KEGG pathway enrichment results showed explicated HLP effect were involved in pathways including inflammatory response according to MAPK signaling pathway, AGE-RAGE signaling pathway in diabetic, IL-17 signaling pathway and TNF signaling pathway, and inflammation was highly related to blood lipid^45^. HLP plays a causal role in the development of CVDs due to endothelial dysfunction^46^, it may be associated with inflammatory response^47^. Meanwhile, dyslipidemia can be significantly improved by ameliorating inflammation^48^. MAPK signaling pathway played a crucial role in inflammation^49^, and participate in various biological processes and the regulation of nuclear factor κB (NF-κB) transcriptional activity. The stimulation of NF- κB increased the levels of downstream inflammatory factors such as TNF-α, IL-1β, and IL-6. MAPKs cascade can transfer extracellular signal to intracellular, thereby causing biological changes such as cell apoptosis^50–53^. AGE-RAGE signaling pathway plays a critical role in recruiting macrophages in inflammation and inducing oxidative stress. Hyperglycemia can activate age-rage signaling pathway, accelerate the synthesis of AGEs, affect the integrity of vascular wall by changing the interaction between matrix and cells, and promote the occurrence and development of vascular and neuropathy. Furthermore, it could lead to many complications of nonalcoholic fatty liver disease, including inflammation, fibrosis and insulin resistance^54,55^. IL-17 signaling pathway is related to the accumulation of proinflammatory chemokines and neutrophils, and plays a role in immune and autoimmune diseases^56^. It has been confirmed that IL-17 can stimulate cell production of TNF-α and IL-6, and TNF-α and IL-6 activate NF-κB by down-regulating TLR2-mediated ERK1/2 phosphorylation, resulting in further effects^57,58^. TNF signaling pathway was an important pathway in inflammation response, TNF activates the canonical NF-κB by regulating TNF receptors and its downstream signaling molecules^59,60^. Hence, we speculated that BSTZC may treat HLP through multiple targets and routes through regulating the level of oxidative stress, inhibiting inflammation and cell apoptosis.

Therefore, IL-6, IL-1β, VEGFA and CASP3 were selected as candidate targets of BSTZC against HLP. In vivo experiment, compared with the control group, the contents of IL-6, IL-1β, VEGFA and CASP3 mRNA were significantly increased (*P* < 0.05, *P* < 0.01) in the model group. These results indicated that HLP can cause the activation of these 4 factors, and their overexpression can lead to inflammatory response and promote cell apoptosis. However, compared with the model group, The quercetin and isorhamnetin groups significantly reduced the elevated levels of IL-6, IL-1B, VEGFA and CASP3 mRNA in model mice (*P* < 0.01). These results indicate that BSTZC can reduce the release of inflammatory mediators and inhibit cell apoptosis, thereby ameliorating HLP. The results of validation test are also consistent with the prediction results of network pharmacology, and it shows that this method has certain accuracy in screening the action target of BSTZC.

## Conclusion

In the current study, an integrated strategy was first developed to explore the core active compounds and molecular mechanisms of BSTZC treats of HLP by network pharmacology analysis and in vivo experimental validation. Quercetin, kaempferol, wogonin, isorhamnetin, baicalein, and luteolin are the core active ingredients of BSTZC, which can regulate most targets related to HLP. Through PPI network screening, we found that 26 core targets including IL6, TNF, VEGFA, CASP3, and IL-1β may provide new ideas for the treatment of HLP. The MAPK signaling pathway, AGE-RAGE signaling pathway in diabetic, IL-17 signaling pathway and TNF signaling pathway were identified as the potential mechanism of BSTZC against HLP. The present work offered the convincing evidence that BSTZC may treat HLP by inhibiting inflammation and apoptosis, and provided the experimental basis for the clinical application of BSTZC in treating HLP (Supplementary Information).

## Supplementary Information

Supplementary Table S1.

## Data Availability

All the data can be obtained from the open-source platform provided in the article, the datasets used and/or analyzed during the current study are available from the corresponding author on reasonable request.
