# Supplementary material for: Network pharmacology analysis and experimental validation to explore the mechanism of Bushao Tiaozhi capsule (BSTZC) on hyperlipidemia
Source: Sci Rep. 2022 Apr 28;12:6992. doi: 10.1038/s41598-022-11139-2 (PMC9051129; doi:10.1038/s41598-022-11139-2)
Supplement: Supplementary file 1 — Supplementary Table S1. [file 41598_2022_11139_MOESM1_ESM.pdf]

Network pharmacology analysis and experimental validation to explore the mechanism of Bushao Tiaozhi Capsule (BSTZC) on hyperlipidemia

Guanlin Xiao[1], Zhihao Zeng[2], Jieyi Jiang[1], Aili Xu[1], Sumei Li[1], Yangxue Li[1], Zhao Chen[1], Weitao Chen[1], Jingnian Zhang[1], Xiaoli Bi [1,2*]

[1] Guangdong Province Engineering and Technology Research Institute of Traditional Chinese Medicine/Guangdong Provincial Key Laboratory of Research and Development in Traditional Chinese Medicine, Guangzhou, 510095, China

[2] School of the Fifth Clinical Medicine, Guangzhou University of Chinese Medicine, Guangzhou, 510405, China

* Corresponding author: Xiaoli Bi, email: zyfxyjs@gzucm.edu.cn

Supplementary Table S1 Bioactive ingredients of BSTZC

No.	MOL ID	Component name	OB (%)	DL	Herb
BZY1	MOL002322	isovitexin	31.29	0.72	BZY
BZY2	MOL000354	isorhamnetin	49.6	0.31	BZY
A	MOL000358	beta-sitosterol	36.91	0.75	BZY/CS
BZY3	MOL000422	kaempferol	41.88	0.24	BZY
BZY4	MOL006331	4',5-Dihydroxyflavone	48.55	0.19	BZY
BZY5	MOL008156	isorhamnetin3-O-D-glucopyranoside_qt	69.01	0.3	BZY
BZY6	MOL000096	(-)-catechin	49.68	0.24	BZY
BZY7	MOL000098	quercetin	46.43	0.28	BZY
CS1	MOL001002	ellagic acid	43.06	0.43	CS
CS2	MOL001918	paeoniflorgenone	87.59	0.37	CS
CS3	MOL001924	paeoniflorin	53.87	0.79	CS
CS4	MOL002714	baicalein	33.52	0.21	CS
CS5	MOL000359	sitosterol	36.91	0.75	CS
CS6	MOL004355	Spinasterol	42.98	0.76	CS
CS7	MOL000449	Stigmasterol	43.83	0.76	CS
CS8	MOL000492	(+)-catechin	54.83	0.24	CS
CS9	MOL006992	(2R,3R)-4-methoxyl-distylin	59.98	0.3	CS
CS10	MOL006999	stigmast-7-en-3-ol	37.42	0.75	CS
CS11	MOL002883	Ethyl oleate (NF)	32.4	0.19	CS
CS12	MOL005043	campest-5-en-3beta-ol	37.58	0.71	CS
EZ1	MOL000296	hederagenin	36.91	0.75	EZ
CXL1	MOL000173	wogonin	30.68	0.23	CXL
CXL2	MOL002928	oroxylin a	41.37	0.23	CXL
CXL3	MOL002932	Panicolin	76.26	0.29	CXL
CXL4	MOL008203	14-deoxy-11-oxo-andrographolide	57.06	0.34	CXL
CXL5	MOL008204	Mono-O-methylwightin	103.11	0.4	CXL
CXL6	MOL008206	Moslosooflavone	44.09	0.25	CXL
CXL7	MOL008209	Deoxycamptothecine	50.01	0.77	CXL
CXL8	MOL008210	Deoxyelephantopin	105.32	0.4	CXL
CXL9	MOL008213	14-deoxy-12-methoxyandrographolide	70.29	0.36	CXL
CXL10	MOL008215	Paniculide B	52.27	0.21	CXL
CXL11	MOL008216	Paniculide C	79.73	0.21	CXL
CXL12	MOL008217	Paniculogenin	47.66	0.75	CXL
CXL13	MOL008219	3-[2-[(1R,4aS,5R,8aS)-5,8a-dimethyl-2-methylene-5-methylol-decalin-1-yl]ethyl]-5H-furan-2-one	51.78	0.28	CXL
CXL14	MOL008222	andrographidine B_qt	72.72	0.33	CXL
CXL15	MOL008226	14-deoxyandrographolide	56.3	0.31	CXL

CXL16	MOL008228	Andrographin	37.57	0.33	CXL
CXL17	MOL008230	andrographidine F_qt	77.13	0.45	CXL
CXL18	MOL008232	(3Z,4S)-3-[2-[(1R,4aS,5R,6R,8aS)-6-hydroxy-5,8a-dimethyl-2-methylene-5-methylol-decalin-1-yl]ethylidene]-4-hydroxy-tetrahydrofuran-2-one	46.96	0.36	CXL
CXL19	MOL008234	andrographolide-19-β-D-glucoside_qt	53.44	0.35	CXL
CXL20	MOL008238	3-[2-[(1S,4aR,5S,8aR)-5,8a-dimethyl-2-methylene-5-methylol-decalin-1-yl]ethyl]-5H-furan-2-one	63.54	0.28	CXL
CXL21	MOL008239	Quercetin tetramethyl(3',4',5,7) ether	31.57	0.41	CXL
CXL22	MOL000006	luteolin	36.16	0.25	CXL

Abbreviations: OB, oral bioavailability; DL, drug-likeness; BZY: Microctis Folium; CS: Paeoniae Radix Rubra; EZ: Curcumae Rhizoma; CXL: Andrographis Herba.

Supplementary Table S2 Detailed biological processes of BSTZC

Catagory	Description	LogP	Count
GO:0062197	cellular response to chemical stress	-23.1003	17
GO:0006979	response to oxidative stress	-19.3567	16
GO:0051090	regulation of DNA-binding transcription factor activity	-17.8524	15
GO:0034599	cellular response to oxidative stress	-18.2974	14
GO:0000302	response to reactive oxygen species	-17.9652	13
GO:0032496	response to lipopolysaccharide	-15.9591	13
GO:0002237	response to molecule of bacterial origin	-15.7406	13
GO:0031667	response to nutrient levels	-13.6373	13
GO:0010038	response to metal ion	-13.749	12
GO:0048732	gland development	-12.8123	12
GO:0050673	epithelial cell proliferation	-12.8123	12
GO:0034614	cellular response to reactive oxygen species	-15.8106	11
GO:0033002	muscle cell proliferation	-14.1058	11
GO:0051091	positive regulation of DNA-binding transcription factor activity	-13.6836	11
GO:0009416	response to light stimulus	-12.8017	11
GO:0009411	response to UV	-14.6793	10
GO:0048660	regulation of smooth muscle cell proliferation	-13.8782	10
GO:0048659	smooth muscle cell proliferation	-13.8264	10
GO:0046686	response to cadmium ion	-13.8017	8
GO:0071276	cellular response to cadmium ion	-13.3760	7
